# Association between organophosphate flame retardant exposure and lipid metabolism: data from the 2013–2014 National Health and Nutrition Examination Survey

**DOI:** 10.3389/fpubh.2024.1340261

**Published:** 2024-03-08

**Authors:** Fu-Jen Cheng, Kai-Fan Tsai, Kuo-Chen Huang, Chia-Te Kung, Wan-Ting Huang, Huey-Ling You, Shau-Hsuan Li, Chin-Chou Wang, Wen-Chin Lee, Hsiu-Yung Pan

**Affiliations:** ^1^Department of Emergency Medicine, Kaohsiung Chang Gung Memorial Hospital, Chang Gung University College of Medicine, Kaohsiung, Taiwan; ^2^Division of Nephrology, Department of Internal Medicine, Kaohsiung Chang Gung Memorial Hospital and Chang Gung University College of Medicine, Kaohsiung, Taiwan; ^3^Department of Laboratory Medicine, Kaohsiung Chang Gung Memorial Hospital and Chang Gung University College of Medicine, Kaohsiung, Taiwan; ^4^Division of Hematology-Oncology, Department of Internal Medicine, Kaohsiung Chang Gung Memorial Hospital and Chang Gung University College of Medicine, Kaohsiung, Taiwan; ^5^Department of Occupational Medicine, Kaohsiung Chang Gung Memorial Hospital and Chang Gung University College of Medicine, Kaohsiung, Taiwan; ^6^Department of Safety, Health and Environmental Engineering, National Kaohsiung University of Science and Technology, Kaohsiung, Taiwan

**Keywords:** organophosphate flame retardants, lipid metabolism, triglycerides, cholesterol, HDL

## Abstract

Organophosphate flame retardants (OPFRs) are emerging environmental pollutants that can be detected in water, dust, and biological organisms. Certain OPFRs can disrupt lipid metabolism in animal models and cell lines. However, the effects of OPFRs on human lipid metabolism remain unclear. We included 1,580 participants (≥20 years) from the 2013–2014 National Health and Nutrition Examination Survey (NHANES) to explore the relationship between OPFR exposure and lipid metabolism biomarkers. After adjusting for confounding factors, results showed that one-unit increases in the log levels of diphenyl phosphate (DPhP) (regression coefficient = −5.755; S.E. = 2.289; *p* = 0.023) and log bis-(1-chloro-2-propyl) phosphate (BCPP) (regression coefficient = −4.637; S.E. = 2.019; *p* = 0.036) were negatively associated with the levels of total cholesterol (TC) in all participants. One-unit increases in the levels of DPhP (regression coefficient = −2.292; S.E. = 0.802; *p* = 0.012), log bis (1,3-dichloro-2-propyl) phosphate (BDCPP) (regression coefficient = −2.046; S.E. = 0.825; *p* = 0.026), and log bis-2-chloroethyl phosphate (BCEP) (regression coefficient = −2.604; S.E. = 0.704; *p* = 0.002) were negatively associated with the levels of high-density lipoprotein cholesterol (HDL-C). With increasing quartiles of urine BDCPP levels, the mean TC levels significantly decreased in all participants (*p* value for trend = 0.028), and quartile increases in the levels of DPhP (*p* value for trend = 0.01), BDCPP (*p* value for trend = 0.001), and BCEP (*p* value for trend<0.001) were negatively corelated with HDL-C, with approximately 5.9, 9.9, and 12.5% differences between the upper and lower quartiles. In conclusion, DPhP, BDCPP, and BCEP were negatively related to HDL-C concentration, whereas DPhP and BCPP levels were negatively associated with TC level. Thus, exposure to OPFRs may interfere with lipid metabolism.

## 1 Introduction

Cardiovascular disease (CVD) is a major leading cause of morbidity and mortality worldwide, and dyslipidemia is an established risk factor for CVD (1). Dyslipidemia is characterized by elevated serum total cholesterol (TC), low-density lipoprotein cholesterol (LDL-C), or triglyceride (TG) levels (2) and reduced serum high-density lipoprotein cholesterol (HDL-C) concentrations. Data from the 2007–2018 National Health and Nutrition Examination Survey (NHANES) showed that the prevalence rates of hypercholesterolemia (TC values ≥ 240 mg/dL) and hypertriglyceridemia (TG levels ≥ 200 mg/dL) were 11.5 and 10.4%, respectively (3). Dyslipidemia can originate from familial disorders (primary) or an alternative underlying etiology, such as metabolic disorder (diabetes, hypothyroidism), medications, unhealthy diet, and poor lifestyle regimen (4).

Organophosphate flame retardants (OPFRs) are ubiquitous in various environmental media because they are physically rather than chemically bound to a material, allowing these compounds to be easily released into the environment (5). Few toxicologic studies have demonstrated that OPFR exposure might interfere with lipid metabolism. TCP exposure might disturb the homeostasis and fluidity of lipid in in cerebrum, spinal cord and sciatic nerve (6). Evidence showed that the meta-isomer of TCP could alter hepatocytes lipid metabolism of seabream through interacting between liver X receptor α and proliferator-activated receptors (PPARs) proteins or modulating the expression levels of micro ribonucleic acids (7). Furthermore, TCP exposure could lead to increased lipid content and alter the fatty acid profile in human hepatocarcinoma (HepG2) cells through activation the pregnane X receptor pathway along with the deficient FA β-oxidation and enhanced lipogenesis (8). Triphenyl phosphate (TPhP, parent compound of diphenyl phosphate) inhibits specific liver carboxylesterases (CEs), altering hepatic lipid metabolism, inducing serum hypertriglyceridemia, and increasing very-low-density lipoprotein (VLDL) and LDL masses in mice (9). TPhP treatment significantly increases blood TC and TG concentrations and induces large lipid droplets in the livers of zebrafish possibly by inhibiting cholesterol utilization and liver lipid transfer (10). Lipid metabolism pathways, such as the fatty acid elongation pathway, are also significantly affected by TPhP exposure (10). TG levels increase and cholesterol levels significantly increase in hepatocytes exposed to high concentrations of tri-m-cresyl phosphate, one of the major isomers of commercial tricresyl phosphate (TCP), in gilthead sea bream (7). Le et al. (11) found that aryl-OPFRs (TPhP and TCP) and chlorinated-OPFRs, such as tris (1,3-dichloro-2-propyl) phosphate (TDCPP), cause lipid accumulation in mouse hepatic cells, accompanied with reduced mitochondrial (mito)-networks/cell, biased mitoATP/glcoATP rate, and expanded mito-area/cell.

Due to dyslipidemia being one of the major risk factors for cardiovascular diseases, toxicological studies have also indicated that exposure to OPFRs may interfere with lipid metabolism. OPFRs have been identified in various environments, including air, dust, water sources, soil, and sediments. Furthermore, traces of OPFRs have been detected in human samples and biotic organisms (12). However, the relationship between OPFRs exposure and lipid metabolism in humans remains unclear. Our study aims to investigate these associations through the analysis of the National Health and Nutrition Examination Survey (NHANES) database.

## 2 Methods

### 2.1 Study population

**Figure 1:**
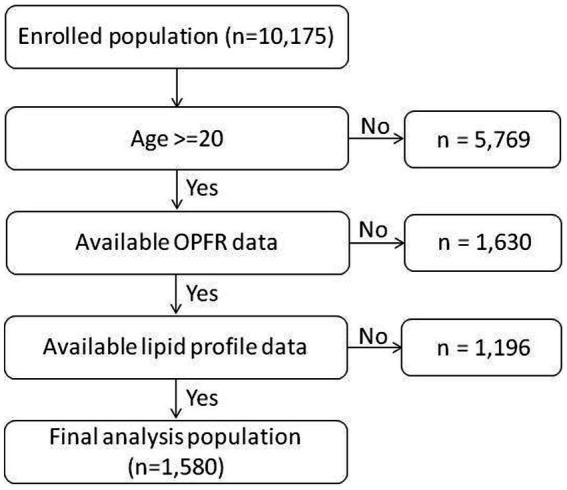
Participant flow chart algorithm.

This study utilized the dataset from the 2013–2014 NHANES dataset in the United States. NHANES is a comprehensive, nationwide, population-based survey initiated in 1999 to evaluate the health and nutritional status of the U.S. population. The 2013–2014 NHANES was in review approved by the US National Center for Health Statistics Research Ethics Review Board (Continuation of Protocol #2011–17), and informed consent was obtained from all participants. The dataset and detailed survey protocols are provided on the NHANES website (13). In the 2013–2014 NHANES, one-third of the participants aged ≥6 years were randomly selected for the measurement of OPFR profiles in stored spot urine samples, and lipid profiles were examined in those who were ≥ 6 years of age and provided serum specimens. In our study, adult participants (≥ 20 years of age) of the 2013–2014 NHANES who had available urinary OPFR profiles and serum lipid data were enrolled for the analysis (*n* = 1,580, Figure 1). We selected the 2013–2014 NHANES data as it includes measurements of both Organophosphate Flame Retardants (OPFRs) and indicators of lipid metabolism.

### 2.2 Measurement of urinary OPFR profiles

The analytical procedure for the urinary OPFR profiles in the 2013–2014 NHANES has been described previously and is available on the NHANES website (13–15). Briefly, a 400 μL urine specimen was utilized for analysis in this study. Analyte extraction involved enzymatic hydrolysis of urinary conjugation followed by solid-phase extraction (SPE) using a 60 mg Strata XAW polymeric sorbent (Phenomenex, Torrance, CA, United States) with 1.5 mL liquid space. The target analytes in the extracts were separated using reversed-phase high-performance liquid chromatography (Agilent, 1,290, Agilent Technologies, Santa Clara, CA, United States) and quantified using isotope dilution-electrospray ionization tandem mass spectrometry (AB Sciex 5,500 Qtrap mass spectrometer, Applied Biosystems, Foster City, CA, United States). In the 2013–2014 NHANES, the following eight OPFR metabolites were measured in the eligible urine samples as exposure surrogates: bis (1-chloro-2-propyl) phosphate (BCPP), bis (1,3-dichloro-2-propyl) phosphate (BDCPP), bis (2-chloroethyl) phosphate (BCEP), diphenyl phosphate (DPhP), di-n-butyl phosphate (DnBP), di-p-cresyl phosphate, di-o-cresyl phosphate, and dibenzyl phosphate. We selected these indicators because previous NHANES data indicated detection rates of BCPP, BDCPP, BCEP, DnBP, and DPhP were above 60%, while other monitored OPFRs in NHANES, such as di-p-cresylphosphate (DpCP), di-o-cresylphosphate (DoCP), dibenzyl phosphate (DBzP), and 2,3,4,5-tetrabromobenzoic acid (TBBA), had detection rates below 20% (15). Therefore, this study investigates the relationship between BCPP, BDCPP, BCEP, DnBP, DPhP, and lipid metabolism. The limits of detection (LODs) were 0.10, 0.11, 0.08, and 0.16 μg/L for BCPP, BDCPP, BCEP, and DPhP, respectively, and 0.05 μg/L for other OPFR metabolites. Urinary metabolites of OPFRs with a detection rate of 50% or higher were considered for statistical analyses. For non-detected samples, a value of LOD/√2 was assigned to estimate urinary concentration during analysis.

### 2.3 Measurement of lipid profiles

At each study site, trained personnel followed the standardized protocol outlined on the NHANES website to collect blood specimens. Detailed procedures for specimen collection are provided in the NHANES Laboratory Procedures Manual (13). The data on TC, LDL-C, HDL-C, and TG were included for analysis in the present study.

### 2.4 Collection of baseline characteristics

In accordance with the NHANES protocols, sociodemographic profiles were collected during household interviews by well-trained interviewers using standardized questionnaires and a computer-assisted personal interview system. Body measurement data were recorded by trained health technicians in NHANES mobile examination centers following standardized procedures. Other procedure details are provided on the NHANES website (13). The age, sex, ethnicity, household income, smoking status, alcohol consumption, and body mass index (BMI) of all participants were recorded.

### 2.5 Statistical analysis

Categorical variables are presented as numbers (*n*) with percentages, and continuous variables are presented as medians with interquartile ranges. To identify baseline covariates associated with lipid profiles and further subgroup analysis, we stratified the study population into subgroups according to age (20–50 vs. >50 years), sex, ethnicity, household income (<4,500 vs. ≥4,500 USD/year), smoking status, alcohol consumption (<12 vs. ≥12 drink/year), and BMI (<25 vs. 25–30 vs. ≥30 kg/m^2^). The lipid profiles were compared between subgroups via the Mann–Whitney U test (for two subgroups) or Kruskal–Wallis H-test (for three or more subgroups). Baseline covariates with a *p*-value of <0.05 in univariate analyses were included in further multivariate analyses for adjustment. We conducted multiple linear regression analyses in the complex samples to explore the relationships between urinary OPFR metabolite concentrations and lipid profiles. The analyses were adjusted for baseline covariates, and sampling weights were applied in accordance with the National Center for Health Statistics Analytic Guidelines. Due to the non-normal distribution, urinary OPFR metabolite concentrations were subjected to logarithmic transformation and quartile stratification before the linear regression analysis. Statistical significance was set at a *p* < 0.05. Statistical Product and Service Solutions (version 22.0; IBM, Armonk, NY, United States) was used for all analyses.

## 3 Results

The serum lipid profiles among different subgroups are listed in Table 1. The median (25, 75 percentile) values of serum TG, TC, LDL-C, and HDL-C among the participants were 118 (80, 190) mg/dL, 187 (160, 215) mg/dL, 107 (87, 132) mg/dL, and 50 (41, 61) mg/dL, respectively. Male (*p* < 0.001), older adults (>50 years, *p* = 0.001), less household income (<4,500 U.S. dollar, *p* = 0.043), and higher BMI (≥30, <0.001) participants had higher levels of TG. Male, older persons, less household income (<4,500 U.S. dollar), and higher BMI (≧25) participants had higher levels of TC. Male, older adults, less household income (<4,500 U.S. dollar), and higher BMI (≥25) participants had lower levels of HDL-C. The concentrations of OPFRs for each subgroup were shown in Supplementary Table S1.

**Table 1:** The median (25 and 75 percentile) of lipid profile in different subgroups.

*N* = 1,580	No	Triglycerides (mg/dL)	*p*	No	Total Cholesterol (mg/dL)	*p*	No	LDL-cholesterol (mg/dL)	*p*	No	Direct HDL-Cholesterol (mg/dL)	*p*
		Median (25, 75%)			Median (25, 75%)			Median (25, 75%)			Median (25, 75%)	
Overall	1,573	118 (80, 190)		1,580	187 (160, 215)		733	107 (87, 132)		1,580	50 (41, 61)	
Sex			<0.001			<0.001			0.530			<0.001
Male	761	131 (87, 213)		763	183 (155, 210)		335	109 (88, 133)		763	45 (37, 53)	
Female	812	112 (76, 166)		817	191 (164, 219)		398	107 (86, 130)		817	55 (46, 68)	
Age (years)			0.001			0.025			0.874			<0.001
20–50	843	110 (76, 189)		847	184 (159, 211)		387	107 (88, 130)		847	48 (40, 59)	
>50	730	127 (88, 191)		733	190 (161, 219)		346	109 (87, 134)		733	51 (42, 65)	
Ethnicity			<0.001			<0.001			0.301			0.001
Mexican-American	214	134 (93, 224)		214	188 (166, 214)		98	110 (91, 129)		214	49 (43, 56)	
Other Hispanic	141	129 (91, 191)		141	193 (165, 221)		63	114 (96, 136)		141	49 (41, 59)	
Non-Hispanic White	715	125 (88, 198)		720	188 (160, 220)		354	108 (87, 134)		720	50 (39, 63)	
Non-Hispanic Black	282	84 (60, 132)		284	175 (149, 175)		120	103 (81, 128)		284	52 (43, 67)	
Other Race – including Multi-Racial	221	118 (83, 223)		221	188 (160, 214)		98	101 (82, 130)		221	49 (40, 60)	
Household income (USD)			0.043			0.023			0.123			<0.001
<4,500	689	123 (84, 198)		692	183 (157, 213)		332	103 (85, 130)		692	48 (40, 59)	
≧4,500	813	114 (78, 182)		817	190 (162, 216)		373	110 (88, 133)		817	51 (42, 63)	
Body mass index (kg/m^2^)			<0.001			0.008			0.296			<0.001
<25	468	95 (68, 136)		470	182 (156, 209)		211	104 (87, 127)		470	56 (46, 70)	
25–30	509	123 (84, 202)		511	188 (163, 217)		252	108 (87, 136)		511	49 (41, 59)	
≧30	587	141 (97, 221)		590	188 (160, 217)		266	110 (89, 133)		590	46 (38, 56)	
Smoking status												
Non-smoker	376	131 (89, 206)	0.843	378	188 (158, 216)	0.574	175	105 (82, 129)	0.75	378	49 (40, 61)	0.091
Current smoker	324	126 (88, 208)		327	183 (160, 215)		145	106 (84, 133)		327	47 (39, 56)	
Alcohol consumption (drink/year)												
<12	1,054	119 (80, 195)	0.34	1,061	186 (159, 215)	0.944	499	107 (86, 131)	0.883	1,061	49 (40, 61)	0.445
≧12	421	118 (78, 178)		421	187 (160, 213)		191	106 (87, 133)		421	50 (42, 62)	

The adjusted regression coefficients (S.E.) for the differences in TG, TC, LDL-C, and HDL-C relative to a one-unit increase in log-transformed DPhP are summarized in Table 2. A one-unit increase in the log DPhP level was negatively associated with TC (regression coefficient = −5.755; S.E. = 2.289; *p* = 0.023) and HDL-C (regression coefficient = −2.292; S.E. = 0.802; *p* = 0.012) in all participants. Subgroup analysis showed that the effects on TC levels (regression coefficient = −9.552; S.E. = 3.506; *p* = 0.016) and HDL-C (regression coefficient = −2.411; S.E. = 1.067; *p* = 0.039) were more prominent in the female group, whereas the effects on HDL-C levels (regression coefficient = −9.552; S.E. = 3.506; *p* = 0.016) were more prominent in the younger group (≤50 years).

**Table 2:** Adjusted regression coefficients (S.E.) for differences in lipid profile relative to a one-unit increase in log10-transformed diphenyl phosphate (DPhP), with results weighted for sampling strategy.

	Triglycerides (mg/dL)			Total Cholesterol (mg/dL)			Direct HDL-Cholesterol (mg/dL)			LDL-cholesterol (mg/dL)		
	Unweighted no./Population size	Regression coefficient (S.E.)	*p*	Unweighted no./Population size	Regression coefficient (S.E.)	*p*	Unweighted no./Population size	Regression coefficient (S.E.)	*p*	Unweighted no./Population size	Regression coefficient (S.E.)	*p*
Overall	1561/220622320	0.843 (4.344)	0.849	1,406/ 203,187,852	−5.775 (2.289)	0.023	1,498 / 213,972,792	−2.292 (0.802)	0.012	731/105236024	−4.500 (2.748)	0.122
Sex												
Male	755/ 106,819,732	16.122 (10.981)	0.163	681/ 98,729,779	−0.237 (5.773)	0.968	719/ 102,840,733	−1.981 (1.003)	0.067	333/ 48,901,316	−0.124 (6.161)	0.984
Female	806/ 113,802,587	−10.299 (5.012)	0.058	725 /104458072	−9.552 (3.506)	0.016	779/ 111,132,059	−2.411 (1.067)	0.039	398/ 56,334,708	−6.912 (4.445)	0.141
Age (years)												
20–50	839/ 124,784,683	12.725 (7.605)	0.115	735/ 112,216,180	−8.272 (4.171)	0.066	807/ 120,910,742	−3.361 (1.263)	0.018	387/ 58,157,597	−4.594 (3.260)	0.179
>50	722/ 95,837,636	−9.246 (6.544)	0.178	671/ 90,971,672	−0.430 (4.665)	0.928	691/ 93,062,050	−0.891 (1.141)	0.447	344/ 47,078,426	−4.861 (4.073)	0.251
Body mass index (kg/m^2^)												
<25	467/ 65,076,848	−1.659 (5.683)	0.774	418/ 59,751,477	−8.133 (3.634)	0.041	450/ 62,953,107	−3.372 (1.778)	0.077	211/ 29,862,967	−2.645 (4.805)	0.59
25–30	508/ 70,207,421	3.103 (11.369)	0.789	454/ 64,078,159	−7.210 (3.845)	0.08	483/ 67,990,603	−2.717 (1.919)	0.177	251/ 36,070,736	−4.292 (5.664)	0.46
≧30	586/ 85,338,049	−0.037 (6.505)	0.995	534/ 79,358,214	−3.301 (6.074)	0.595	565/ 83,029,082	−1.152 (1.244)	0.369	265/ 38,950,494	−7.275 (5.472)	0.204
Smoking status												
Non-smoker	371/ 53,265,487	−2.750 (9.917)	0.785	347/ 51,129,953	−3.583 (9.139)	0.701	362/ 52,515,709	−1.266 (1.289)	0.342	174/ 25,693,514	0.600 (8.855)	0.947
Current smoker (reference)	323/ 42,288,856	13.000 (17.924)	0.479	289/ 38,719,205	−6.613 (5.262)	0.228	313/ 41,401,542	−1.304 (2.432)	0.6	145/ 18,532,400	−6.179 (6.065)	0.324
Alcohol consumption (drink/year)												
<12	1,048/ 157,895,769	4.311 (5.287)	0.428	1,014/ 154,537,025	−7.710 (3.571)	0.047	1,014/ 154,537,025	−2.794 (1.056)	0.018	498/ 76,573,828	−7.637 (4.077)	0.081
≧12	415/ 50,927,354	−10.455 (8.453)	0.235	392/ 48,650,827	−1.507 (4.479)	0.741	392/ 48,650,827	−1.023 (1.556)	0.521	190/ 23,446,728	1.379 (3.500)	0.7
Income												
< 4,500	684/ 79,624,539	−9.092 (9.695)	0.363	645/ 75,642,861	−7.352 (4.221)	0.102	687/ 79,877,915	−0.638 (1.018)	0.54	331/ 38,366,303	−7.679 (2.341)	0.005
≧4,500	807/ 133,310,219	8.794 (9.612)	0.375	761/ 127,544,990	−4.528 (2.179)	0.055	811/ 134,094,877	−3.301 (0.914)	0.003	372/ 64,320,395	−1.818 (3.886)	0.647
Ethnicity												
Mexican-American	213/ 19,744,438	−7.864 (21.619)	0.723	184/ 17,049,107	−6.727 (5.359)	0.238	193/ 18,020,950	0.844 (2.414)	0.733	98/ 8,920,471	−6.272 (6.096)	0.338
Other Hispanic	141/ 13,089,856	42.381 (28.189)	0.157	122/ 11,323,919	0.242 (9.151)	0.979	130/ 12,232,075	−0.779 (1.199)	0.527	63/ 5,394,797	−11.990 (10.227)	0.279
Non-Hispanic White	707/ 145,840,898	−4.860 (5.508)	0.392	671/ 138,431,591	−4.836 (2.718)	0.095	694/ 143,134,248	−2.630 (0.734)	0.003	353/ 73,074,926	−3.539 (3.627)	0.345
Non-Hispanic Black	279/ 24,290,277	18.664 (9.565)	0.073	246/ 21,266,181	−6.655 (4.350)	0.15	271/ 23,603,730	−3.140 (1.941)	0.13	119/ 10,150,633	−12.266 (4.940)	0.03
Other Race – including Multi-Racial	221/ 17,656,849	6.163 (11.926)	0.613	183/ 15,117,052	−11.975 (9.327)	0.219	210/ 16,981,787	−2.715 (2.577)	0.309	98/ 7,695,195	−1.614 (6.800)	0.817

HDL, high-density lipoprotein; LDL, low-density lipoprotein; SE, standard error.

The adjusted regression coefficients (S.E.) for the differences in TG, TC, LDL-C, and HDL-C relative to a one-unit increase in log-transformed BDCPP are summarized in Table 3. We found that a one-unit increase in the log BDCPP level was negatively associated with the levels of HDL-C (regression coefficient = −2.046; S.E. = 0.825; *p* = 0.026). Subgroup analysis showed that the effects on TC levels (regression coefficient = −8.559; S.E. = 2.616; *p* = 0.005) were more prominent in the female group, whereas the effects on HDL-C levels were more prominent in the male (regression coefficient = −2.455; S.E. = 1.044; *p* = 0.033), older participant (>50 years, regression coefficient = −3.596; S.E. = 1.108; *p* = 0.005), and non-Hispanic White (>50 years, regression coefficient = −2.334; S.E. = 0.894; *p* = 0.02) groups.

**Table 3:** Adjusted regression coefficients (S.E.) for differences in TG, cholesterol, LDL, and HDL relative to a one-unit increase in log_10_-transformed biomarkers of bis(1,3-dichloro-2-propyl) phosphate (BDCPP), with results weighted for sampling strategy.

	Triglycerides (mg/dL)			Total Cholesterol (mg/dL)			Direct HDL-Cholesterol (mg/dL)			LDL-cholesterol (mg/dL)		
	Unweighted no./Population size	Regression coefficient (S.E.)	*p*	Unweighted no./Population size	Regression coefficient (S.E.)	*p*	Unweighted no./Population size	Regression coefficient (S.E.)	*p*	Unweighted no./Population size	Regression coefficient (S.E.)	*p*
Overall	1547/218730635	−4.065 (4.906)	0.42	1,392 / 201,296,167	−3.492 (2.293)	0.149	1,484 / 212,081,107	−2.046 (0.825)	0.026	723 (103619237)	3.264 (2.651)	0.237
Sex												
Male	746/ 105,454,411	0.254 (11.564)	0.983	672/ 97,364,458	1.975 (3.018)	0.523	710/ 101,475,412	−2.455 (1.044)	0.033	329/ 47,884,816	8.214 (4.771)	0.106
Female	801/ 113,276,224	−9.175 (4.878)	0.08	720/ 103,931,709	−8.559 (2.616)	0.005	774/ 110,605,695	−1.599 (1.184)	0.197	394/ 55,734,421	−0.599 (2.808)	0.834
Age (years)												
20–50	836/ 124,381,639	−8.599 (9.064)	0.358	732/ 111,813,136	−3.480 (3.975)	0.395	804/ 120,507,698	−0.517 (1.081)	0.639	384/ 57,607,126	0.196 (3.978)	0.961
>50	711/ 94,348,995	3.213 (8.379)	0.707	660/ 89,483,031	0.253 (4.124)	0.952	680/ 91,573,409	−3.596 (1.108)	0.005	339/ 46,012,111	7.613 (2.959)	0.021
Body mass index (kg/m^2^)												
<25	466/ 65,056,262	−7.985 (6.237)	0.22	417/ 59,730,890	−10.621 (4.408)	0.029	449/ 62,932,520	−3.991 (1.876)	0.05	210/ 29,666,207	5.848 (4.143)	0.179
25–30	505/ 70,016,577	3.046 (8.641)	0.729	451/ 63,887,315	−6.374 (3.944)	0.127	480/ 67,799,758	−2.764 (1.543)	0.093	250/ 36,024,585	−2.165 (4.512)	0.638
≧30	576/ 83,657,796	−8.449 (14.360)	0.565	524/ 77,677,960	3.134 (4.992)	0.54	555/ 81,348,828	−0.091 (1.117)	0.936	259/ 37,576,618	5.164 (3.912)	0.207
Smoking status												
Non-smoker	364/ 52,321,024	−4.731 (12.254)	0.705	340/ 50,185,491	−0.525 (4.486)	0.908	355/ 51,571,246	−0.638 (1.694)	0.712	171/ 24,902,281	10.388 (3.677)	0.013
Current smoker	322/ 42,092,096	13.018 (18.495)	0.492	288/ 38,522,446	1.049 (5.026)	0.837	312/ 41,204,783	−1.896 (2.400)	0.442	144/ 18,335,641	2.806 (4.213)	0.515
Alcohol consumption (drink/year)												
<12	1,039/ 156,380,798	−0.082 (6.187)	0.99	1,005 (153022054)	−3.845 (3.481)	0.287	1,005/ 153,022,054	−2.600 (0.966)	0.017	493/ 75,162,700	3.217 (3.632)	0.39
≧12	410/ 50,550,640	−15.300 (10.005)	0.147	387 (48274113)	−1.857 (4.515)	0.687	387/ 48,274,113	−1.047 (1.727)	0.554	187/ 23,241,070	4.844 (4.629)	0.314
Income												
<4,500	675/ 78,653,374	−0.295 (6.556)	0.965	636/ 74,671,696	−6.524 (3.311)	0.068	678/ 78,906,750	−1.040 (1.335)	0.448	326/ 37,701,516	3.774 (2.450)	0.144
≧4,500	802/ 132,389,700	−4.467 (8.505)	0.607	756/ 126,624,471	−1.778 (3.080)	0.572	806/ 133,174,357	−2.712 (1.148)	0.032	369/ 63,368,396	2.921 (3.481)	0.415
Ethnicity												
Mexican-American	209/ 19,545,948	15.489 (13.578)	0.278	180/ 16,850,617	0.311 (8.565)	0.972	189/ 17,822,460	−1.765 (2.286)	0.456	97/ 8,865,799	1.474 (8.061)	0.86
Other Hispanic	140/ 12,988,838	−15.480 (17.761)	0.399	121/ 11,222,901	−1.103 (13.522)	0.936	129/ 12,131,057	2.719 (2.460)	0.289	62/ 5,293,780	−10.639 (6.302)	0.135
Non-Hispanic White	700/ 144,473,908	−6.160 (6.337)	0.346	664/ 137,064,601	−2.598 (2.787)	0.366	687/ 141,767,259	−2.334 (0.894)	0.02	347/ 71,652,013	5.515 (3.262)	0.112
Non-Hispanic Black	278/ 24,133,897	4.338 (5.944)	0.478	245/ 21,109,801	−9.441 (5.528)	0.111	270/ 23,447,350	−4.486 (3.590)	0.233	119/ 10,112,448	−6.816 (3.992)	0.116
Other Race – including Multi-Racial	220/ 17,588,041	−12.028 (10.159)	0.255	182/ 15,048,245	−10.255 (7.130)	0.171	209/ 16,912,979	−0.597 (1.885)	0.756	98/ 7,695,195	−6.816 (5.890)	0.272

HDL, high-density lipoprotein; LDL, low-density lipoprotein; SE, standard error.

The adjusted regression coefficients (S.E.) for the differences in TG, TC, LDL-C, and HDL-C relative to a one-unit increase in the log-transformed BCPP level are summarized in Table 4. A one-unit increase in the log BCPP level was negatively associated with TC levels (regression coefficient = −4.637; S.E. = 2.019; *p* = 0.036). Subgroup analysis also showed the negative association of BCPP level with the levels of TG (regression coefficient = −13.286; S.E. = 5.626; *p* = 0.032) and TC (regression coefficient = −9.410; S.E. = 3.448; *p* = 0.016) in the female group.

**Table 4:** Adjusted regression coefficients (S.E.) for differences in TG, cholesterol, LDL, and HDL relative to a one-unit increase in log10-transformed biomarkers of bis-(1-chloro-2-propyl) phosphate (BCPP), with results weighted for sampling strategy.

	Triglycerides (mg/dL)			Total Cholesterol (mg/dL)			Direct HDL-Cholesterol (mg/dL)			LDL-cholesterol (mg/dL)		
	Unweighted no./Population size	Regression coefficient (S.E.)	*p*	Unweighted no./Population size	Regression coefficient (S.E.)	*p*	Unweighted no./Population size	Regression coefficient (S.E.)	*p*	Unweighted no./Population size	Regression coefficient (S.E.)	*p*
Overall	1564/221207358	−10.195 (6.605)	0.144	1,409 / 203,772,890	−4.637 (2.019)	0.036	1,501 / 214,557,830	−1.302 (0.962)	0.196	733 (105576082)	0.938 (1.967)	0.64
Sex												
Male	757/ 107,159,790	−8.520 (10.973)	0.45	683/99069837	−0.424 (4.919)	0.932	721/ 103,180,791	−1.012 (1.057)	0.354	335/ 49,241,374	2.420 (4.238)	0.576
Female	807/ 114,047,567	−13.286 (5.626)	0.032	726/ 104,703,052	−9.410 (3.448)	0.016	780/ 111,377,039	−1.376 (1.625)	0.41	398/ 56,334,708	−0.656 (3.449)	0.852
Age (years)												
20–50	840/ 125,029,663	−16.135 (8.700)	0.083	736/ 112,461,159	−5.568 (2.611)	0.05	808/ 121,155,721	−0.470 (1.320)	0.727	387/ 58,157,597	−2.797 (2.291)	0.241
>50	724/ 96,177,694	0.693 (10.362)	0.948	673/ 91,311,730	−1.835 (3.935)	0.648	693/ 93,402,109	−2.482 (1.594)	0.14	346/ 47,418,484	6.926 (3.641)	0.077
Body mass index (kg/m^2^)												
< 25	468/ 65,321,828	−9.355 (10.087)	0.368	419/ 59,996,457	−7.060 (4.152)	0.11	451/ 63,198,087	−1.049 (2.519)	0.683	211/ 29,862,967	3.520 (4.175)	0.412
25–30	509/ 70,510,125	−27.472 (8.951)	0.008	455/ 64,380,864	−8.508 (5.872)	0.168	484/ 68,293,307	−2.064 (1.431)	0.17	252/ 36,373,441	−1.266 (4.764)	0.794
≧30	587/ 85,375,403	4.860 (11.891)	0.689	535/ 79,395,568	−0.011 (5.288)	0.998	566/ 83,066,435	−0.696 (1.414)	0.63	266/ 38,987,848	0.285 (6.649)	0.966
Smoking status												
Non-smoker	373/ 53,547,820	−17.431 (14.932)	0.261	349/ 51,412,287	−8.019 (5.211)	0.145	364/ 52,798,042	−0.164 (0.902)	0.859	175/ 25,730,867	−0.876 (5.645)	0.879
Secondhand smoke												
Current smoker	323/ 42,288,856	−6.269 (21.363)	0.773	289/ 38,719,205	−6.192 (5.965)	0.316	313/ 41,401,542	−1.888 (2.035)	0.368	145/ 18,532,400	7.312 (4.860)	0.153
Alcohol consumption (drink/year)												
<12	1,050/ 158,178,103	−8.849 (7.042)	0.228	1,016/ 154,819,358	−5.194 (1.931)	0.017	1,016/ 154,819,358	−1.578 (0.869)	0.089	499/ 76,611,182	−0.252 (2.138)	0.908
≧12	416/ 51,230,058	−18.495 (13.987)	0.206	393/ 48,953,531	−3.646 (5.984)	0.551	393/ 48,953,531	−1.006 (2.683)	0.713	191/ 23,749,432	4.675 (7.369)	0.537
Income												
<4,500	685/ 79,661,893	−11.414 (9.587)	0.252	646/ 75,680,215	−3.627 (3.708)	0.344	688/ 79,915,269	0.566 (0.992)	0.577	332/ 38,403,657	3.905 (4.846)	0.433
≧4,500	809/ 133,857,903	−10.892 (9.769)	0.282	763/ 128,092,674	−5.318 (2.414)	0.044	813/ 134,642,561	−2.233 (1.308)	0.109	373/ 64,623,099	−1.149 (2.369)	0.635
Ethnicity												
Mexican-American	213/ 19,744,438	−23.376 (13.074)	0.101	184/ 17,049,107	−7.905 (4.079)	0.081	193/ 18,020,950	0.550 (1.708)	0.753	98/ 8,920,471	10.110 (6.244)	0.149
Other Hispanic	141/ 13,089,856	13.511 (36.005)	0.714	122/ 11,323,919	4.941 (7.113)	0.5	130/ 12,232,075	−0.355 (2.522)	0.89	63/ 5,394,797	−0.973 (5.076)	0.853
Non-Hispanic White	709/ 146,388,582	−17.209 (8.371)	0.058	673/ 138,979,276	−8.009 (3.103)	0.021	696/ 143,681,933	−1.321 (1.368)	0.35	354/ 73,377,630	−0.541 (2.586)	0.837
Non-Hispanic Black	280/ 24,327,630	16.231 (6.510)	0.027	247/ 21,303,534	6.561 (5.413)	0.247	272/ 23,641,084	−1.621 (3.954)	0.688	120/ 10,187,986	−1.492 (5.812)	0.802
Other Race – including Multi-Racial	221/ 17,656,849	13.031 (9.382)	0.185	183/ 15,117,052	8.366 (6.667)	0.229	210/ 16,981,787	−2.657 (1.878)	0.178	98/ 7,695,195	12.590 (6.103)	0.064

HDL, high-density lipoprotein; LDL, low-density lipoprotein; SE, standard error.

The adjusted regression coefficients (S.E.) for the differences in TG, TC, LDL-C, and HDL-C relative to a one-unit increase in the log-transformed BCEP level are summarized in Table 5. A one-unit increase in the log BCEP level was negatively associated with the levels of HDL-C (regression coefficient = −2.604; S.E. = 0.704; *p* = 0.002), especially among the groups of female (regression coefficient = −2.959; S.E. = 1.003; *p* = 0.01), BMI less than 25 (regression coefficient = −4.449; S.E. = 1.259; *p* = 0.003), non-smoker (regression coefficient = −3.209; S.E. = 1.315; *p* = 0.028), less alcohol consumption (<12 drinks per year, regression coefficient = −3.423; S.E. = 0.881; *p* = 0.001), household income more than 4,500 USD (regression coefficient = −3.485; S.E. = 0.806; *p* = 0.001), and non-Hispanic white (regression coefficient = −3.307; S.E. = 0.850; *p* = 0.001).

**Table 5:** Adjusted regression coefficients (S.E.) for differences in TG, cholesterol, LDL, and HDL relative to a one-unit increase in log10-transformed biomarkers of bis-2-chloroethyl phosphate (BCEP), with results weighted for sampling strategy.

	Triglycerides (mg/dL)			Total cholesterol (mg/dL)			Direct HDL-cholesterol (mg/dL)			LDL-cholesterol (mg/dL)		
	Unweighted no./Population size	Regression coefficient (S.E.)	*p*	Unweighted no./Population size	Regression coefficient (S.E.)	*p*	Unweighted no./Population size	Regression coefficient (S.E.)	*p*	Unweighted no./Population size	Regression coefficient (S.E.)	*p*
Overall	1558/220639307	3.530 (5.042)	0.495	1,404/ 203,314,871	−3.828 (3.043)	0.228	1,496 / 214,099,811	−2.604 (0.704)	0.002	730/ 105,308,960	−4.816 (2.985)	0.127
Sex												
Male	754/ 106,869,064	6.313 (8.560)	0.472	680/ 98,779,111	−0.392 (4.950)	0.938	718/ 102,890,065	−2.071 (0.993)	0.054	334/ 49,141,545	−0.553 (4.855)	0.911
Female	804/ 113,770,243	1.191 (4.639)	0.801	724/ 104,535,759	−6.730 (2.139)	0.007	778/ 111,209,746	−2.959 (1.003)	0.01	396/ 56,167,415	−8.562 (3.517)	0.028
Age (years)												
20–50 (1)	835/ 124,561,441	8.025 (5.500)	0.165	732/ 112,102,969	−2.418 (2.882)	0.415	804/ 120,797,531	−2.493 (0.823)	0.008	385/ 57,990,304	−3.713 (4.163)	0.387
>50 (2)	723/ 96,077,866	−3.346 (8.561)	0.701	672/ 91,211,901	−6.323 (3.610)	0.1	692/ 93,302,280	−2.869 (1.320)	0.046	345/ 47,318,655	−6.210 (3.110)	0.064
Body mass index (kg/m^2^)												
<25	466/ 65,123,302	7.016 (7.029)	0.334	417/ 59,797,931	−4.501 (3.095)	0.166	449/ 62,999,561	−4.449 (1.259)	0.003	210/ 29,763,138	−0.713 (2.887)	0.808
25–30	508/ 70,417,925	0.641 (11.185)	0.955	454/ 64,288,664	−12.173 (3.678)	0.005	483/ 68,201,107	−3.148 (1.624)	0.072	252/ 36,373,441	−10.635 (3.670)	0.011
≧30	584/ 85,098,079	4.892 (6.512)	0.464	533/ 79,228,275	2.742 (5.134)	0.601	564/ 82,899,142	−1.119 (0.829)	0.197	264/ 38,820,555	−2.363 (4.506)	0.608
Smoking status												
Non-smoker	372/ 53,461,416	−6.359 (8.573)	0.47	348/ 51,325,882	−5.973 (7.420)	0.433	363/ 52,711,637	−3.209 (1.315)	0.028	174/ 25,644,463	−7.838 (4.814)	0.124
Current smoker	323/ 42,288,856	23.285 (14.926)	0.14	289/ 38,719,205	7.999 (5.712)	0.182	313/ 41,401,542	−1.661 (1.543)	0.299	145/ 18,532,400	0.232 (5.177)	0.965
Alcohol consumption (drink/year)												
<12	1,047/ 157,882,970	7.826 (4.973)	0.136	1,014/ 154,634,256	−3.488 (3.886)	0.383	1,014/ 154,634,256	−3.423 (0.881)	0.001	498/ 76,524,777	−6.432 (3.477)	0.084
≧12	413/ 50,957,142	−10.917 (9.275)	0.257	390/ 48,680,615	−4.473 (3.733)	0.249	390/ 48,680,615	−0.265 (1.287)	0.839	189/ 23,568,715	0.404 (3.547)	0.911
Household income												
<4,500	684/ 79,581,005	2.471 (6.229)	0.697	645/ 75,599,327	−3.742 (2.160)	0.104	687/ 79,834,381	−1.149 (0.734)	0.138	331/ 38,322,768	−3.446 (1.998)	0.105
≧4,500	805/ 133,480,773	3.852 (10.378)	0.716	759/ 127,715,544	−3.896 (4.070)	0.354	809/ 134,265,430	−3.485 (0.806)	0.001	371/ 64,436,866	−5.451 (4.371)	0.231
Ethnicity												
Mexican-American	213/ 19,744,438	−6.630 (11.457)	0.574	184/ 17,049,107	−4.665 (4.034)	0.274	193/ 18,020,950	0.169 (1.485)	0.911	98/ 8,920,471	−0.882 (3.984)	0.831
Other Hispanic	140/ 13,003,452	40.215 (26.208)	0.149	121/ 11,237,514	10.681 (9.506)	0.281	129/ 12,145,670	2.265 (1.698)	0.205	62/ 5,308,393	−9.262 (6.981)	0.226
Non-Hispanic white	708/ 146,288,754	1.165 (6.707)	0.864	672/ 138,879,447	−5.250 (4.144)	0.224	695/ 143,582,104	−3.307 (0.850)	0.001	353/ 73,277,802	−4.704 (3.700)	0.223
Non-Hispanic black	276/ 23,945,813	16.027 (12.845)	0.234	244/ 21,031,748	12.918 (4.488)	0.013	269/ 23,369,298	−1.499 (2.417)	0.546	119/ 10,107,098	−0.602 (7.067)	0.934
Other Race – including multi-racial	221/ 17,656,849	6.173 (13.214)	0.647	183/ 15,117,052	−14.219 (8.038)	0.097	210/ 16,981,787	−2.692 (1.899)	0.177	98/ 7,695,195	−10.415 (3.943)	0.023

HDL, high-density lipoprotein; LDL, low-density lipoprotein; SE, standard error.

The adjusted regression coefficients (S.E.) for the differences in TG, TC, LDL-C, and HDL-C levels relative to a one-unit increase in the log-transformed DnBP level (μg/L) are summarized in Table 6. The association between a one-unit increase in the log DnBP level and TG, TC, LDL-C, and HDL-C levels did not achieve statistical significance in the overall group.

**Table 6:** Adjusted regression coefficients (S.E.) for differences in triglycerides, cholesterol, HDL-cholesterol, and LDL-cholesterol relative to a one-unit increase in log10-transformed di-n-butyl phosphate (DnBP), with results weighted for sampling strategy.

	Triglycerides (mg/dL)			Total Cholesterol (mg/dL)			Direct HDL-Cholesterol (mg/dL)			LDL-cholesterol (mg/dL)		
	Unweighted no./Population size	Regression coefficient (S.E.)	*p*	Unweighted no./Population size	Regression coefficient (S.E.)	*p*	Unweighted no./Population size	Regression coefficient (S.E.)	*p*	Unweighted no./Population size	Regression coefficient (S.E.)	*p*
Overall	1562/220901865	−4.959 (8.188)	0.554	1407/203467397	−3.116 (3.353)	0.367	1499/214252337	−2.160 (1.174)	0.086	733/105576082	−0.688 (3.377)	0.841
Sex												
Male	755/106854298	−1.692 (12.696)	0.896	681/98764345	−4.288 (4.135)	0.316	719/102875298	−2.505 (1.465)	0.108	335/49241374	−0.315 (6.245)	0.96
Female	807 (114047567)	−11.228 (8.393)	0.201	726/104703052	−1.805 (4.582)	0.699	780/111377039	−1.725 (1.672)	0.319	398/56334708	−1.148 (6.071)	0.853
Age (years)												
20–50	838/124724170	−4.826 (13.054)	0.717	734/112155666	0.612 (3.103)	0.846	806/120850228	−0.927 (1.487)	0.542	387/58157597	0.631 (4.106)	0.88
>50	724/96177694	0.456 (12.388)	0.971	673/91311730	−4.627 (5.507)	0.414	693/93402109	−3.340 (1.885)	0.097	346/47418484	−2.094 (5.211)	0.694
Body mass index (kg/m^2^)												
<25	468/65321828	−5.214 (12.084)	0.672	419/59996457	−3.042 (5.048)	0.556	451/63198087	−3.752 (1.448)	0.02	211/29862967	9.576 (6.545)	0.164
25–30	509/70510125	2.979 (19.754)	0.882	455/64380864	−10.542 (3.560)	0.01	484/68293307	−2.926 (2.569)	0.273	252/36373441	−6.324 (6.713)	0.361
≧30	585/85069910	−12.723 (18.083)	0.492	533/79090075	2.396 (6.956)	0.735	564/82760942	0.004 (1.677)	0.998	266/38987848	−4.756 (5.435)	0.395
Smoking status												
Non-smoker	373/53547820	−16.561 (11.746)	0.179	349/51412287	−11.878 (6.878)	0.105	364/52798042	−0.773 (1.721)	0.66	175/25730867	−3.321 (10.078)	0.746
Current smoker	322/42192883	4.778 (21.176)	0.825	288/38623233	8.177 (10.375)	0.443	312/41305570	−1.698 (2.421)	0.494	145/18532400	−3.434 (9.813)	0.731
Alcohol consumption (drink/year)												
<12	1049/158082130	1.395 (11.438)	0.905	1015/154723386	−5.180 (3.694)	0.181	1015/154723386	−3.669 (1.415)	0.02	499/76611182	−3.720 (4.935)	0.463
≧12	415/51020538	−16.084 (9.472)	0.11	392/48744011	1.912 (5.566)	0.736	392/48744011	1.010 (1.689)	0.558	191/23749432	5.667 (6.934)	0.429
Income												
< 4,500	684/79565920	−6.880 (8.443)	0.428	645/75584243	0.941 (5.287)	0.861	687/79819296	−0.541 (1.539)	0.73	332/38403657	1.728 (4.227)	0.688
≧4,500	808/133648383	−1.855 (9.590)	0.849	762/127883154	−5.500 (3.587)	0.146	812/134433040	−3.162 (1.448)	0.045	373/64623099	−1.725 (4.107)	0.68
Ethnicity												
Mexican-American	213/19744438	−15.340 (22.589)	0.511	184/17049107	3.033 (6.246)	0.638	193/18020950	1.725 (2.251)	0.46	98/8920471	14.337 (9.700)	0.183
Other Hispanic	141/13089856	−29.299 (19.973)	0.166	122/11323919	−0.908 (7.730)	0.908	130/12232075	2.791 (1.792)	0.143	63/5394797	−12.621 (11.122)	0.294
Non-Hispanic White	707/146083089	−2.618 (9.358)	0.784	671/138673782	−5.388 (4.532)	0.253	694/143376440	−2.624 (1.451)	0.091	354/73377630	−4.179 (4.696)	0.388
Non-Hispanic Black	280/24327630	5.526 (10.495)	0.607	247/21303534	10.385 (5.589)	0.086	272/23641084	−1.477 (2.663)	0.589	120/10187986	4.265 (5.953)	0.489
Other Race – including Multi-Racial	221/17656849	5.273 (19.514)	0.791	183/15117052	−5.324 (10.904)	0.632	210/16981787	−5.455 (3.191)	0.108	98/7695195	10.506 (5.173)	0.067

HDL, high-density lipoprotein; LDL, low-density lipoprotein; SE, standard error.

**Figure 2:**
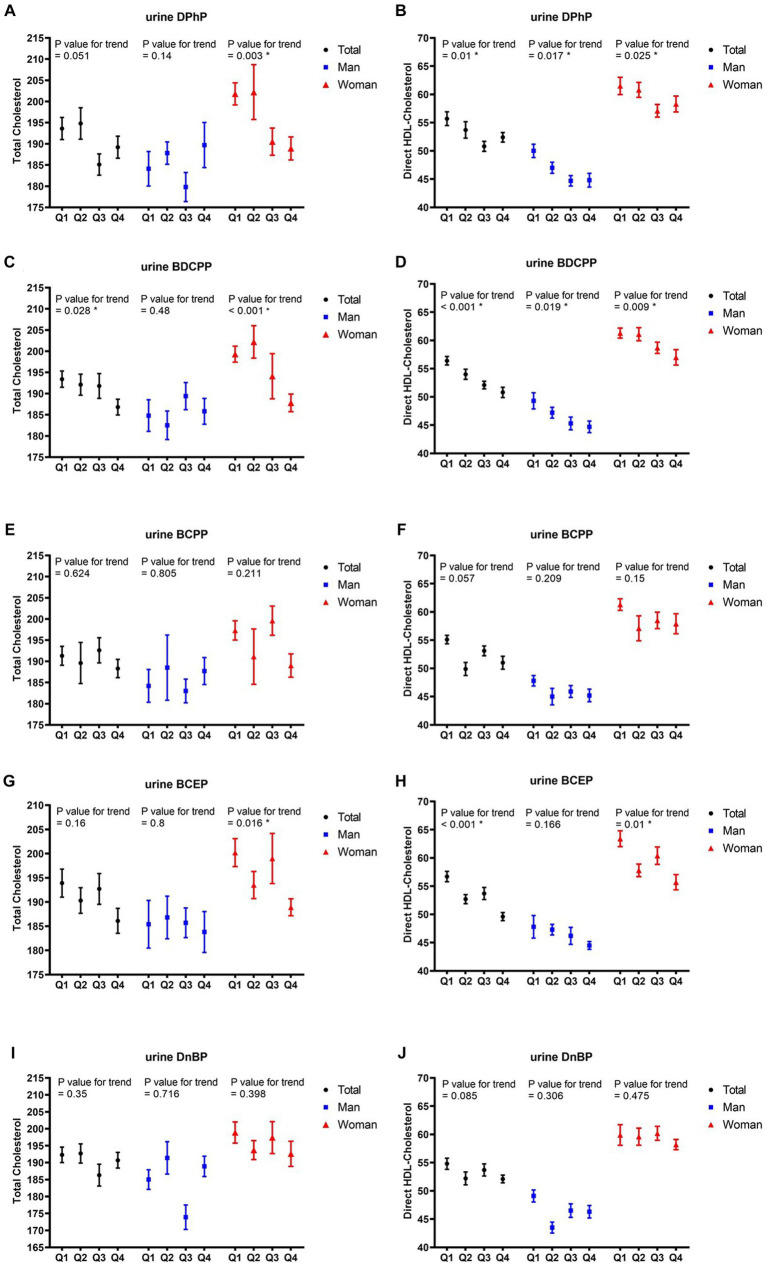
Mean and SE of cholesterol and HDL, across quartiles of OPFRs in linear regression models, with results weighted for sampling strategy. **(A,B)** Diphenyl phosphate (DPhP), **(C,D)** bis(1,3-dichloro-2-propyl) phosphate (BDCPP), **(E,F)** bis(1-chloro-2-propyl) phosphate (BCPP), **(G,H)** bis(2-chloroethyl) phosphate (BCEP), and **(I,J)** di-n-butyl phosphate (DnBP). HDL, high-density lipoprotein; SE, standard error.

After adjusting for potential covariates in multiple regression analysis, the correlations between the quartiles of each OPFR and TC, as well as HDL-C in all participants and different sexes are listed in Figure 2. With increasing quartiles of urine BDCPP levels, the mean TC levels significantly decreased in all participants (*p* value for trend = 0.028) and the female group (*p* value for trend<0.001), whereas the mean differences in TC levels between the upper and lower quartiles of BDCPP in all participants and the female group were 3.4 and 5.8%, respectively. Furthermore, the quartile increase in urine BCEP level was negatively related to TC levels (*p* value for trend = 0.016) in the female group, with approximately 5.6% difference between the upper and lower quartiles. Quartile increases in the levels of DPhP (*p* value for trend = 0.01), BDCPP (*p* value for trend = 0.001), and BCEP (*p* value for trend<0.001) were negatively corelated with HDL-C, with approximately 5.9, 9.9, and 12.5% differences between the upper and lower quartiles. Conversely, we also observed gender differences in the impact of OPFRs on HDL-C. In males, DPhP (*p* value for trend = 0.017) and BDCPP (*p* value for trend = 0.019) levels were negatively correlated with HDL-C levels, with a decrease of approximately 10.4 and 9.3%, respectively, in the highest quartile of HDL-C compared with the lowest quartile. In contrast to the male group, the female group showed negative correlations of DPhP (*p* value for trend = 0.025), BDCPP (*p* value for trend = 0.009), and BCEP (*p* value for trend = 0.01) with HDL-C. The highest quartile of DPhP, BDCPP, and BCEP levels in the females was associated with approximately 5.2, 7.0, and 12.1% reductions in HDL-C, respectively, compared with the lowest quartile.

## 4 Discussion

In the present study, we observed a statistically significant association between OPFRs and lipid profiles. After adjusting for confounding factors, the DPhP level was negatively associated with TC and HDL-C levels, the BDCPP level was negatively associated with HDL-C levels, the BCPP level was negatively associated with TC levels, and the BCEP level was negatively associated with HDL-C levels. Furthermore, quartile increases in the levels of DPhP, BDCPP, and BCEP were negatively correlated with HDL-C, with approximately 5.9, 9.9, and 12.5% differences between the upper and lower quartiles.

Dyslipidemia is one of major risk factors for cardiovascular and cerebrovascular diseases, leading to an increased risk of atherosclerotic cardiovascular disease (16). Among lipid metabolites, HDL-C has been found to be associated with mortality. Li et al. followed 7,766 older adults individuals aged ≥65 years, and found that the group with HDL-C < 61 mg/dL had higher rates of all-cause mortality and cardiovascular-related mortality (17). Another research also indicated that for each 1 mg/dL increase in HDL-C, there is a 3.7 to 4.7% decrease in the rate of cardiovascular mortality (18). In our study, we observed a negative correlation between the levels of DPhP, BDCPP, and BCEP with HDL-C. This may suggest that populations with higher levels of DPhP, BDCPP, and BCEP could potentially have an increased risk of cardiovascular diseases. Further research is needed to clarify this association.

Recently, a few studies have investigated the effects of exposure to OPFRs on fatty acid metabolism. Hu et al. (19) found that the exposure of RAW264.7 macrophage cells to TPhP increases endoplasmic reticulum (ER) stress and inflammation, which further downregulate and decrease fatty acid saturation. Lpcat3 is one of the factors that regulate carbohydrate metabolism and adipocyte differentiation. Another study using Alpha mouse liver 12 cells found that exposure to two aryl-OPFRs (TCP and TPhP) and three chlorinated OPFRs (TDCPP, TCPP, and TCEP) causes intracellular lipid accumulation at relatively low concentrations (<10 μmol/L) for TCP, TPHP, and TDCPP. They also observed intracellular lipid accumulation at concentrations >10 μmol/L for TCPP and TCEP. This study also found that OPFRs increase oxidative stress and alter mitochondrial membrane potential in liver cells, thereby interfering with ATP metabolism and causing lipid accumulation (11). Meanwhile, CEs are responsible for hydrolyzing xenobiotic or endogenous compounds that contain ester, thioester, or amide groups (20). In the liver, CEs are responsible for metabolizing TGs and fatty acids in lipid droplets and resynthesizing them into VLDL in the ER, which is then released into the bloodstream, further affecting the metabolism of carbohydrates and esters and promoting insulin resistance (21). In a previous study, exposure to TPhP inhibits CE activity in the liver of mice, resulting in increased concentrations of LDL-C and VLDL in the serum (9). The reason for the inhibition of CEs may be that OPFRs irreversibly bind to the activation site of CEs, thereby inhibiting their function. Another study using Atlantic cod liver found that exposure to TCPP, 2-ethyldiphenyl phosphate, or their mixture downregulates the expression of genes involved in cholesterol synthesis and affects subsequent lipid metabolism (22). Cholesterol is a precursor for steroid hormones, such as follicle-stimulating hormone, luteinizing hormone, total testosterone, and total estradiol, and interference of cholesterol synthesis might lead to endocrine disruption. However, limited studies focused on the relationship between OPFR exposure and human lipid metabolism. The results of the present study showed that DPhP, BDCPP, and BCEP were negatively related to HDL-C, whereas DPhP and BCPP were negatively associated with TC. In addition, OPFRs exerted differential effects on lipid metabolism interference in males and females. Specifically, the negative correlation of DPhP, BDCPP, and BCEP with TC was more pronounced in females than in males. However, the association between OPFRs exposure and HDL-C showed less gender difference. Further investigation is warranted to clarify the reasons for this gender difference.

OPFRs are low-cost and effective flame retardants widely used in various consumer products, building materials, textiles, and electronics. They have been used to replace polybrominated diphenyl ethers (PBDEs) owing to the persistence, bioaccumulation, and toxicity of the latter. By 2011, OPFRs accounted for 20% of the global flame retardant market (23). They are also used as plasticizers for epoxy resins, coatings, engineering thermoplastics, and floor polishes. The consumption of OPFRs reached 83,000 tons in Europe and 72,000 tons in the United States in 2007, and the usage has grown at a rate of 3.7% annually from 2007 to 2012 (24). However, OPFRs are physically rather than chemically bound to the products, allowing them to easily detach from the products during use and enter the surrounding environment through volatilization, dissolution, deposition, and infiltration. OPFRs can be detected in various environmental and biological matrices, such as air (25), soil (26), water (27), fish (28), and even breast milk (29). Humans can be exposed to OPFRs through skin contact, inhalation, and ingestion (30). OPFRs with low logarithmic octanol-air coefficient values (Log K_oa_) mainly exist in the gas phase; hence, the contribution of these compounds in the air is greater than that in the dust. Exposure to volatile OPFRs such as TCPP and TCEP usually occurs through air inhalation. By contrast, OPFRs with high Log K_oa_ are primarily in the particulate phase and settled on dust. Therefore, dust ingestion is the important exposure pathway for less volatile OPRFs, such as tris (2-butoxyethyl) phosphate (TBEP), TCP, and TPhP. A study from Vietnam reported that the total estimated daily intakes of ΣOPFRs via dermal absorption, air inhalation, and dust ingestion for toddlers and adults under medial exposure are 160 ngkg^−1^ day^−1^ and 36.7 gkg^−1^ day^−1^, respectively (31). The value is approximately 4–5 times greater in toddlers than in adults. Dermal absorption is the major exposure pathway for toddlers and adults (accounting for 45.1 and 49.5% ΣOPFRs, respectively), followed by air inhalation (contributing to 40 and 46.5% ΣOPFRs, respectively). Human OPFR exposure could be estimated by studying the concentration of OPFRs and their metabolites in bio-samples, such as urine, serum, semen, breast milk, and hair. Urine is a commonly used biomatrix because its collection is easy and noninvasive. OPFR metabolites in urine are linked to external OPFR exposure; for instance, the urinary concentration of DPhP, a metabolite of TPhP, has been associated with TPhP in handwipes and dust (32). Data extracted from the 2013–2014 NHANES showed that BDCIPP and DPhP were present in approximately 92% of the participants, BCEP in 89%, DnBP in 81%, and BCPP in 61% of the US general population (15). Among the OPFRs studied, DPhP had the highest concentration range (<0.16–193 μg/L), followed by BDCPP (<0.11–169 μg/L) and BCEP (<0.08–110 μg/L). Bis (1-chloro-2-propyl) 1-hydroxy-2-propyl phosphate (a metabolite of TCPP) and DPHP were the most frequently detected compounds in urine (detection frequency > 98%) and the most abundant compounds in urine, accounting for 46% (median level 720 pg./mL) and 39% (medial level 610 pg./mL) of ΣOPFRs, respectively, in one study from Norway (12). The detection frequencies in urine were greater than 90% for DPhP, DnBP (metabolites of tri-n-butyl phosphate), bis-(2-butoxyethyl) phosphate (a metabolite of TBEP), and dicresyl phosphate (a metabolite of TCP), with relatively low detection frequencies of BDCPP (76%) and BCEP (71%) in southern China (33). Among all OPRFs investigated in the present study, DPhP (0.55 ng/mL) exhibited the highest mean level, followed by BCEP (0.72 ng/mL), DnBP (0.29 ng/mL), and BCPP (0.094 ng/mL). The overall urinary detection rate of OPFRs was 98.8% in a chronic kidney disease population in Taiwan (34). In the present study, the detection rate and median level were 78.31% and 0.134 μg/g creatinine (Cr) for DPhP, 78.31% and 0.212 μg/g Cr for TBEP, 64.46% and 0.025 μg/g Cr, and 59.64% and 0.186 μg/g Cr for BBOEP, respectively. Universal exposure to OPFRs was proved by growing evidence, and the disturbance of OPFR exposure on lipid metabolism might result in more and more adverse health impacts.

Dyslipidemia is an important risk factor for atherosclerotic CVD and ischemic cerebrovascular accident (CVA). Insulin resistance, which is associated with metabolic syndrome, increases plasma TG and LDL-C levels and reduces HDL-C levels, thereby increasing the risk for atherosclerotic CVD, CVA, and peripheral artery disease. High-density lipoproteins are involved in delaying the formation of atherosclerotic lesions through several mechanisms, such as removal of cholesterol from macrophages within the arterial wall and transportation to the liver for excretion (35, 36). Observational studies found that a 1 mg/dL (0.026 mmol/L) increase in HDL-C is associated with a 3% risk reduction of coronary heart disease in women and 2% risk reduction in men, irrespective of age, body mass index, smoking habit, blood pressure, and LDL-C level (16). In a nationwide, community-based, prospective cohort study in the US, the risk for all-cause mortality was significantly higher in the group with HDL-C concentrations <61 mg/dL than in the group with HDL-C concentrations ranging from 61 to 87 mg/dL among older adults (aged ≥65 years). Repeatedly measured low HDL-C levels (defined as <40 mg/dL for men and < 50 mg/dL for women) have been associated with the risk of thyroid cancer, and this correlation is stronger in metabolically unhealthy Korean persons (37). Data from a large German primary care provider database showed that low HDL-C concentrations (<40 mg/dL) are positively associated and elevated TC levels (>200 mg/dL) are negatively associated with cancer, irrespective of diabetes, obesity, age, and sex. By contrast, serum levels of TG and LDL pose no impact on cancer risk (38). In the present study, exposure to DPhP, BDCPP, and TCEP were negatively associated with HDL-C. However, whether these negative associations result in adverse health outcome merits further investigation.

The majority of total cellular cholesterol is localized on the plasma membranes and interacts with the adjacent lipids to regulate the rigidity, fluidity, and permeability of the cell membrane. Cholesterol could bind to numerous transmembrane proteins and either maintain or alter their conformation. It can also interact with several transport proteins that facilitate cholesterol trafficking and regulate the subcellular distribution. In addition to their roles in membrane structure and function, derivatives of cholesterol are engaged in various biological processes, such as steroid hormone generation and bile acid production. The homeostasis of cholesterol is determined by *de novo* biosynthesis, uptake, export, and storage (39). Negative associations of DPhP and BCPP levels with TC levels were disclosed in our study. TBEP exposure in Tm3 Leydig cells increases oxidative stress, decreases cell viability, disrupts hormone generation (40), and induces abnormal sperm morphology and testicular histopathology in male rats (41). Moreover, TPhP and TDCPP can cause endocrine disruption, alter thyroid hormone levels (42), and decrease semen quality in men (43). DPhP downregulates the expression of genes involved in lipid/cholesterol and glucose/fatty acid metabolism (44). An animal study revealed that exposure to DPHP causes metabolic disturbance in the organism possibly because of its interfering effects on estrogen and mineralocorticoids (45). Thyroid hormone is an important regulator of serum cholesterol levels and hepatic cholesterol metabolism, including synthesis, endocytosis by the (LDL)-receptor, and peripheral uptake and hepatic excretion by reverse cholesterol transport. The disruption of cholesterol metabolism by OPFRs might further interfere with thyroid hormone synthesis.

There are several limitations about our study. First, the composition and concentration of different OPFRs might be varied in different regions, hence, the results might not be applied to other countries. Second, several kinds of chemicals such as phthalates and polybrominated diphenylethers are co-existing in the environment. These chemicals might interfere with OPRFs which lead to different impacts on human health. The interactions between different environmental toxicants and its effects on human health could not be further clarified in our study. Third, the mechanisms of lipid metabolism might vary between different persons biochemically, therefore, the disturbance from OPFRs on lipid metabolism might also be different. The concomitant medical illness and medications might also exert different degrees of influence of lipid metabolism which could not be delineated in our study.

## 5 Conclusion

DPhP, BDCPP, and TCEP levels were negatively related to the concentrations of HDL-C, whereas DPhP and BCPP levels were negatively associated with the levels of total cholesterol. Furthermore, the mean differences in TC levels between the upper and lower quartiles of BDCPP in all participants and the female group were 3.4 and 5.8%, respectively. Conversely, quartile increases in DPhP, BDCPP, and BCEP levels were negatively corelated with HDL-C levels, with approximately 5.9, 9.9, and 12.5% differences between the upper and lower quartiles. The findings of the current study may suggest that exposure to OPFRs could potentially interfere with lipid metabolism and have associated health effects.

## Data availability statement

The original contributions presented in the study are included in the article/supplementary material, further inquiries can be directed to the corresponding author.

## Ethics statement

The studies involving humans were approved by the 2013–2014 NHANES and by the US National Center for Health Statistics Research Ethics Review Board (Continuation of Protocol #2011-17), and informed consent was obtained from all participants. The studies were conducted in accordance with the local legislation and institutional requirements. The human samples used in this study were acquired from another research group. Written informed consent for participation was not required from the participants or the participants’ legal guardians/next of kin in accordance with the national legislation and institutional requirements.

## Author contributions

F-JC: Writing – original draft, Writing – review & editing, Conceptualization, Methodology, Formal analysis, Validation. K-FT: Formal analysis, Resources, Software, Validation, Writing – original draft. K-CH: Data curation, Investigation, Writing – review & editing. C-TK: Project administration, Resources, Supervision, Writing – review & editing. W-TH: Resources, Writing – review & editing. H-LY: Validation, Writing – review & editing. S-HL: Software, Visualization, Writing – review & editing. C-CW: Data curation, Writing – review & editing. W-CL: Investigation, Validation, Writing – review & editing. H-YP: Conceptualization, Data curation, Investigation, Supervision, Validation, Visualization, Writing – original draft, Writing – review & editing.

## Glossary

CVD: Cardiovascular disease
TC: total cholesterol
LDL-C: low-density lipoprotein cholesterol
HDL-C: high-density lipoprotein cholesterol
TG: triglyceride
NHANES: National Health and Nutrition Examination Survey
OPFRs: organophosphate flame retardants
TPhP: triphenyl phosphate
CEs: carboxylesterases
VLDL: very-low-density lipoprotein
TDCPP: tris (1,3-dichloro-2-propyl) phosphate
SPE: solid-phase extraction
BCPP: bis (1-chloro-2-propyl) phosphate
BDCPP: bis (1,3-dichloro-2-propyl) phosphate
BCEP: bis (2-chloroethyl) phosphate
DPhP: diphenyl phosphate
BMI: body mass index
ER: endoplasmic reticulum
TBEP: tris (2-butoxyethyl) phosphate
PBDEs: polybrominated diphenyl ethers
PBDEs: polybrominated diphenyl ethers
CVA: cerebrovascular accident
LODs: limits of detection
Lpcat3: lysophosphatidylcholine acyltransferase 3
