# Supplementary material for: Association between organophosphate flame retardant exposure and lipid metabolism: data from the 2013–2014 National Health and Nutrition Examination Survey
Source: Front Public Health. 2024 Mar 8;12:1340261. doi: 10.3389/fpubh.2024.1340261 (PMC10959188; doi:10.3389/fpubh.2024.1340261)
Supplement: Supplementary file 1 [file Table_1.docx]

**Supplementary Material**

Table S1. Mean and SE of cholesterol and HDL, across quartiles of OPFRs in linear regression models, with results weighted for sampling strategy.

|  | Total Cholesterol (mean, SE) | | | Direct HDL-Cholesterol (mean, SE) | | |
| --- | --- | --- | --- | --- | --- | --- |
|  | Total | Man | Woman | total | Man | Woman |
| **urine DPhP** |  |  |  |  |  |  |
| Q1 | 193.6 (2.606) | 184.1 (4.074) | 201.8 (2.607) | 55.7 (1.217) | 50.0 (1.172) | 61.5 (1.526) |
| Q2 | 194.8 (3.719) | 187.8 (2.638) | 202.2 (6.467) | 53.7 (1.457) | 47.0 (0.978) | 60.8 (1.326) |
| Q3 | 185.1 (2.510) | 179.8 (3.411) | 190.5 (3.201) | 50.8 (0.887) | 44.7 (0.916) | 57.1 (1.123) |
| Q4 | 189.2 (2.600) | 189.7 (5.325) | 188.9 (2.721) | 52.4 (0.851) | 44.8 (1.215) | 58.3 (1.406) |
| P value for trend | 0.051 | 0.14 | 0.003 | 0.01 | 0.017 | 0.025 |
| **urine BDCPP** |  |  |  |  |  |  |
| Q1 | 193.4 (1.929) | 184.8 (3.727) | 199.3 (1.890) | 56.4 (0.756) | 49.3 (1.419) | 61.3 (0.893) |
| Q2 | 192.1 (2.484) | 182.5 (3.362) | 202.2 (3.825) | 54.0 (0.899) | 47.2 (0.959) | 61.1 (1.162) |
| Q3 | 191.8 (2.928) | 189.4 (3.197) | 194.1 (5.335) | 52.1 (0.668) | 45.3 (1.129) | 58.7 (1.001) |
| Q4 | 186.8 (1.856) | 185.8 (3.048) | 187.8 (2.076) | 50.8 (0.912) | 44.7 (1.032) | 57.0 (1.375) |
| P value for trend | 0.028 | 0.48 | <0.001 | <0.001 | 0.019 | 0.009 |
| **urine BCPP** |  |  |  |  |  |  |
| Q1 | 191.3 (2.234) | 184.2 (3.871) | 197.3 (2.287) | 55.1 (0.743) | 47.8 (0.926) | 61.3 (1.032) |
| Q2 | 189.6 (4.842) | 188.5 (7.688) | 191.1 (6.544) | 49.9 (1.155) | 45.0 (1.449) | 57.1 (2.211) |
| Q3 | 192.6 (2.967) | 183.0 (2.795) | 199.6 (3.442) | 53.1 (0.871) | 45.9 (1.054) | 58.5 (1.458) |
| Q4 | 188.3 (2.158) | 187.7 (3.186) | 189.0 (2.748) | 51.0 (1.144) | 45.2 (1.117) | 57.9 (1.776) |
| P value for trend | 0.624 | 0.805 | 0.211 | 0.057 | 0.209 | 0.15 |
| **urine BCEP** |  |  |  |  |  |  |
| Q1 | 193.9 (2.900) | 185.4 (4.914) | 200.2 (2.884) | 56.7 (0.919) | 47.8 (1.994) | 63.4 (1.398) |
| Q2 | 190.3 (2.639) | 186.8 (4.395) | 193.5 (2.813) | 52.7 (0.804) | 47.3 (0.930) | 57.8 (1.114) |
| Q3 | 192.7 (3.194) | 185.7 (3.063) | 199.0 (5.179) | 53.7 (1.069) | 46.2 (1.510) | 60.4 (1.541) |
| Q4 | 186.1 (2.577) | 183.8 (4.224) | 188.9 (1.760) | 49.6 (0.715) | 44.5 (0.695) | 55.7 (1.353) |
| P value for trend | 0.16 | 0.8 | 0.016 | <0.001 | 0.166 | 0.01 |
| **urine DnBP** |  |  |  |  |  |  |
| Q1 | 192.3 (2.279) | 185.0 (2.886) | 198.9 (3.118) | 54.8 (0.991) | 49.1 (1.065) | 59.9 (1.819) |
| Q2 | 192.7 (2.834) | 191.4 (4.781) | 193.7 (2.792) | 52.2 (1.138) | 43.5 (0.967) | 59.6 (1.506) |
| Q3 | 186.3 (3.211) | 173.9 (3.606) | 197.4 (4.721) | 53.7 (1.071) | 46.5 (1.196) | 60.2 (1.232) |
| Q4 | 190.7 (2.321) | 188.9 (3.004) | 192.6 (3.722) | 52.1 (0.678) | 46.3 (1.099) | 58.2 (0.901) |
| P value for trend | 0.35 | 0.716 | 0.398 | 0.085 | 0.306 | 0.475 |

HDL, high-density lipoprotein; SE, standard error; DPhP, diphenyl phosphate; BDCPP, bis(1,3-dichloro-2-propyl) phosphate; BCPP, bis(1-chloro-2-propyl) phosphate; BCEP, bis(2-chloroethyl) phosphate, DnBP, di-n-butyl phosphate
